# Role of the intestinal microbiota and diet in the onset and progression of colorectal and breast cancers and the interconnection between both types of tumours

**DOI:** 10.20517/mrr.2023.36

**Published:** 2023-11-27

**Authors:** Sergio Ruiz-Saavedra, Aida Zapico, Sonia González, Nuria Salazar, Clara G. de los Reyes-Gavilán

**Affiliations:** ^1^Department of Microbiology and Biochemistry of Dairy Products, Instituto de Productos Lácteos de Asturias (IPLA-CSIC), Villaviciosa 33300, Spain.; ^2^Diet, Microbiota and Health Group, Instituto de Investigación Sanitaria del Principado de Asturias (ISPA), Oviedo 33011, Spain.; ^3^Department of Functional Biology, University of Oviedo, Oviedo 33006, Spain.

**Keywords:** Gut microbiota, diet, colorectal cancer, breast cancer, dietary fibre, polyphenol

## Abstract

Colorectal cancer (CRC) is among the leading causes of mortality in adults of both sexes worldwide, while breast cancer (BC) is among the leading causes of death in women. In addition to age, gender, and genetic predisposition, environmental and lifestyle factors exert a strong influence. Global diet, including alcohol consumption, is one of the most important modifiable factors affecting the risk of CRC and BC. Western dietary patterns promoting high intakes of xenobiotics from food processing and ethanol have been associated with increased cancer risk, whereas the Mediterranean diet, generally leading to a higher intake of polyphenols and fibre, has been associated with a protective effect. Gut dysbiosis is a common feature in CRC, where the usual microbiota is progressively replaced by opportunistic pathogens and the gut metabolome is altered. The relationship between microbiota and BC has been less studied. The estrobolome is the collection of genes from intestinal bacteria that can metabolize oestrogens. In a dysbiosis condition, microbial deconjugating enzymes can reactivate conjugated-deactivated oestrogens, increasing the risk of BC. In contrast, intestinal microorganisms can increase the biological activity and bioavailability of dietary phytochemicals through diverse microbial metabolic transformations, potentiating their anticancer activity. Members of the intestinal microbiota can increase the toxicity of xenobiotics through metabolic transformations. However, most of the microorganisms involved in diet-microbiota interactions remain poorly characterized. Here, we provide an overview of the associations between microbiota and diet in BC and CRC, considering the diverse types and heterogeneity of these cancers and their relationship between them and with gut microbiota.

## INTRODUCTION

Colorectal (CRC) and breast cancer (BC) present the highest incidence rates in Europe (12.1% and 11.8%, respectively), ranking among the leading causes of mortality in both sexes in the case of CRC and in women for BC^[1]^.

Approximately 80% of CRC cases are developed through the conventional adenoma pathway^[2]^ and originate from mucus-producing intestinal cells^[3,4]^. The adenoma-carcinoma sequence in the CRC progression usually begins with the development of abnormal structures of a benign nature, known as polyps or adenomas, in the epithelial tissue^[5]^. Subsequent activation of oncogenes and inactivation of tumour suppressor genes in polyps frequently lead to dysplastic lesions and the transformation of these structures into adenocarcinomas. Malignant adenocarcinomas spread further and invade nearby tissues, even penetrating all four intestinal histological layers^[3,5]^. Subsequently, the tumours may disseminate to other organs and metastasize via the lymphatic or haematogenous route^[3]^. Approximately 20% of CRC cases develop through the serrated pathway^[6]^. In the last decades, serrated lesions have gained clinical attention as serrated cases show distinctive endoscopic, anatomopathological, and molecular signatures from conventional adenomas. Hyperplastic polyps are the serrated lesions that are associated with a lower risk of turning malignant, accounting for 80% of cases, while sessile serrated adenomas (20% of serrated cases), accompanied by dysplasia or not, show a higher risk of turning into CRC and its surgical removal is always recommended^[7]^.

BC is a heterogeneous disease with different molecular subtypes from an immunohistochemical perspective that relies on the expression of oestrogen receptor (ER), progesterone receptor (PR), and human epidermal growth factor receptor 2 (HER2). BC positive for oestrogen receptors (ER+), progesterone receptors (PR+), or both (ER+/PR+) is considered Hormone Receptor-Positive (HR) BC. To date, four major invasive breast carcinoma genetic subtypes from a clinical point of view have been identified with prognostic and therapeutic relevance. These are: the luminal subtype A presenting high expression of ER and PR receptors without HER2 overexpression and low cell proliferation index, the luminal subtype B (ER+, PR±, HER2±, and high proliferation index), HER2 enriched tumours (ER-/PR- and HER2+), and finally the triple-negative BC subgroup or TNBC (ER-/PR- and HER2-)^[8]^. BC was the leading cause of cancer mortality for women worldwide in 2020^[1]^ and is classified into different grades and types based on histological characteristics. The histological grade is a well-established prognostic tool based on the degree of aggressiveness or differentiation of the tumour tissue, including a combined score for several parameters such as the microscopic evaluation of tubule or gland formation, nuclear pleomorphism, and the mitotic count (i.e., determination of the proliferation marker Ki-67 by immunohistochemistry). In addition, the TNM is a recognized classification to designate the BC stage at the time of diagnosis and refers to the size and invasiveness of the tumour (T), lymph node involvement (N), and the presence of distant metastases (M)^[9]^. The intra-epithelial neoplasia of ductal carcinoma is considered a pre-invasive lesion at risk for invasive cancer^[10]^. Invasive ductal carcinoma is one of the most common types of BC (80% of the cases), whereas invasive lobular carcinoma accounts for 5%-15% of BC cases and a mixed type of both carcinomas is present in 3%-5% of cases^[11]^.

### Factors affecting the development of CRC and BC

Several factors such as age, genetic predisposition, and environment influence the risk of these non-communicable diseases. Among them, age is one of the most significant, as the risk of spontaneous CRC and BC in the general population increases progressively from the age of 50 years^[2,12,13]^.

Another major factor is genetic predisposition, as 10% of the processes are related to hereditary CRC syndromes^[14]^ such as MUTYH gene-associated polyposis (MAP), familial adenomatous polyposis (FAP) or hereditary non-polyposis-associated CRC (Lynch syndrome)^[12]^ and the family history of CRC. In this regard, first-degree family history doubles or triples the risk of CRC^[15]^. In the case of BC, around 5%-10% of the cases are hereditary, 50% of which are due to deleterious mutations in high (*BRCA1*, *BRCA2*, *TP53*, *PTEN*, *STK11*, *CDH1*, *PALB2*) or moderate penetrance genes (*CHEK2*, *ATM*, *BRIP1*, *BARD1*, *RAD51C*, and *RAD51D*)^[13]^. In addition, gender (higher incidence in men for CRC and women for BC), origin (higher incidence in individuals of African origin for both cancers), or the presence of other health conditions increase the risk for both tumours^[12,15-18]^.

Environmental and lifestyle factors influence CRC risk, which could be on the basis of the increasing incidence of CRC in developed countries^[19,20]^. Some modifiable risk factors for cancer depend mainly on the individual’s lifestyle (diet, alcohol or tobacco consumption, *etc*.) and health practises (such as cholecystectomy, radiotherapy, or breast screening practices)^[12,15,17,18]^. The association between smoking and CRC or BC remains controversial and depends on the time of exposure, age of onset, and the amount consumed (number of cigarettes per day)^[13,15]^. Regarding the consumption of alcoholic beverages, analyses indicate that the intake of more than two alcoholic beverages per day doubles the risk for CRC^[15]^. Global diet is also one of the major players in the development of CRC^[21]^. Epidemiological evidence on the effect of specific dietary factors pointed to red and processed meats as potential carcinogens, with special emphasis on alcohol consumption, which has been consistently associated with an increased risk of BC and CRC^[22]^.

In addition, in BC, variables concerning women’s reproductive history, such as the age at menarche and at first birth, parity, breast density, chest irradiation, breastfeeding habits, and use of oral contraceptives, have an impact on disease development^[23,24]^. The effect of the body mass index (BMI) on the risk of developing BC changes according to women’s reproductive status. While the postmenopausal years are directly related to an increased risk, in the childbearing years, the tendency is slightly protective^[24]^.

### The connection between BC and CRC in women

Recent studies have supported the existence of a connection between BC and CRC in women^[25]^. BC patients present a 60% increased risk of developing CRC^[26]^, and although no consensus has been achieved, the elevated levels of endogenous/exogenous sex hormones due to hormone/oestrogen replacement therapies and BC treatments (tamoxifen) may be possible factors contributing to increasing the risk of colorectal tumorigenesis^[25]^. Higher dietary consumption of potentially carcinogenic compounds such as heterocyclic amines (HCAs) and lower bioactives such as fibres may modulate gut microbiota towards a proinflammatory status^[27-29]^. Although inflammation and dysbiosis alone may not be sufficient to promote tumorigenesis, complex interactions between established risk factors for cancer, including genetic predisposition, obesity, dietary intake, and alcohol consumption, seem to alter the gut microbiota and contribute to carcinogenesis^[30]^. Diet has proven to be key in the modulation of gut microbiota in short- and long-term dietary interventions by providing substrates that can differentially promote the growth of specific microorganisms in the colon^[31]^. Dietary fibre and polyphenols are compounds with proven prebiotic effects that contribute to this modulation^[32]^. Dietary fibre is not digested in the gastrointestinal tract, but it is fermented in the colon by the gut microbiota, leading to the generation of short-chain fatty acids (SCFAs) such as acetate, butyrate, and propionate^[32]^. Microbiota contributes to the maintenance of the overall health status of an individual. Microorganisms not only participate in the metabolic degradation of indigestible carbon sources such as fibres, but also exert a protective inhibition of pathogen adhesion to intestinal surfaces and trophic maintenance of the intestinal epithelium integrity and functionality^[27]^. The aim of this review was to analyse the role and association between microbiota and diet in the onset and progression of both processes, BC and CRC. This can provide a holistic approach to the interconnection between both types of cancer and the influence exerted on them by the binomial microbiota-diet, aspects that are not frequently addressed together.

### Gut-Brain axis dysregulation by chemotherapy

Chemotherapy is part of cancer care for a variety of tumours. Drug cytotoxicity impacts the central nervous system, leading to neuroinflammation and damage to the blood-brain barrier. It is also known that chemotherapy causes significant disruption of the intestinal microbiota^[33,34]^. The vagus nerve provides innervation to the gastrointestinal tract and acts as a direct route of communication of the microbiota-gut-brain axis, serving as a modulator of this circuit in inflammatory and psychiatric disorders. In addition, the hypothalamic-pituitary-adrenal axis, which coordinates the neuroendocrine response to stress, is involved in microbiota-gut-brain communication^[35]^. The non-selective nature of chemotherapy drugs implies that they also target some non-malignant cells. The parts of the body most susceptible to this toxicity are the gastrointestinal tract, the central nervous system, and the communication between them. Consequently, some adverse symptoms due to chemotherapy include sickness, diarrhoea and vomiting, anxiety and depression, fatigue, pain, and cognitive impairments. Healthcare professionals have underestimated, particularly, the chemotherapy-induced cognitive impairment over the years. As a result, this complication remains under-reported and poorly understood by clinicians and researchers. Focusing on the role of the microbiota on these side-adverse effects, it is possible that microbial disruption due to chemotherapy causes changes to vagal afferent signalling and disrupts the hypothalamic-pituitary-adrenal axis, which may impact mood, cognitive function, and other alterations related to central nervous and immunity systems.

## DIETARY PATTERNS LINKED TO THE RISK OF CANCER DEVELOPMENT

Diet is one of the modifiable lifestyle factors that mainly contribute to the occurrence and severity of some human pathologies, such as cancer. Despite extensive research in the past few years, no consensus on the role of dietary factors in cancer prevention has been reached. The fact that tumour development is a long-term process makes the identification of cause-effect relationships difficult. Humans are exposed to a mixture of substances through food consumption, and evaluating their interaction through *in vivo* studies proves challenging. On one hand, food provides mutagens, but it also serves as a source of bioactive compounds, mainly derived from plant foods. Therefore, the understanding of the net effect of diet on health depends on the global balance between all the factors involved. Rather than analysing the relationships between the consumption of each food and the risk of the pathology, some authors suggest considering the dietary and lifestyle patterns into which each compound falls^[16]^.

**Figure 1:**
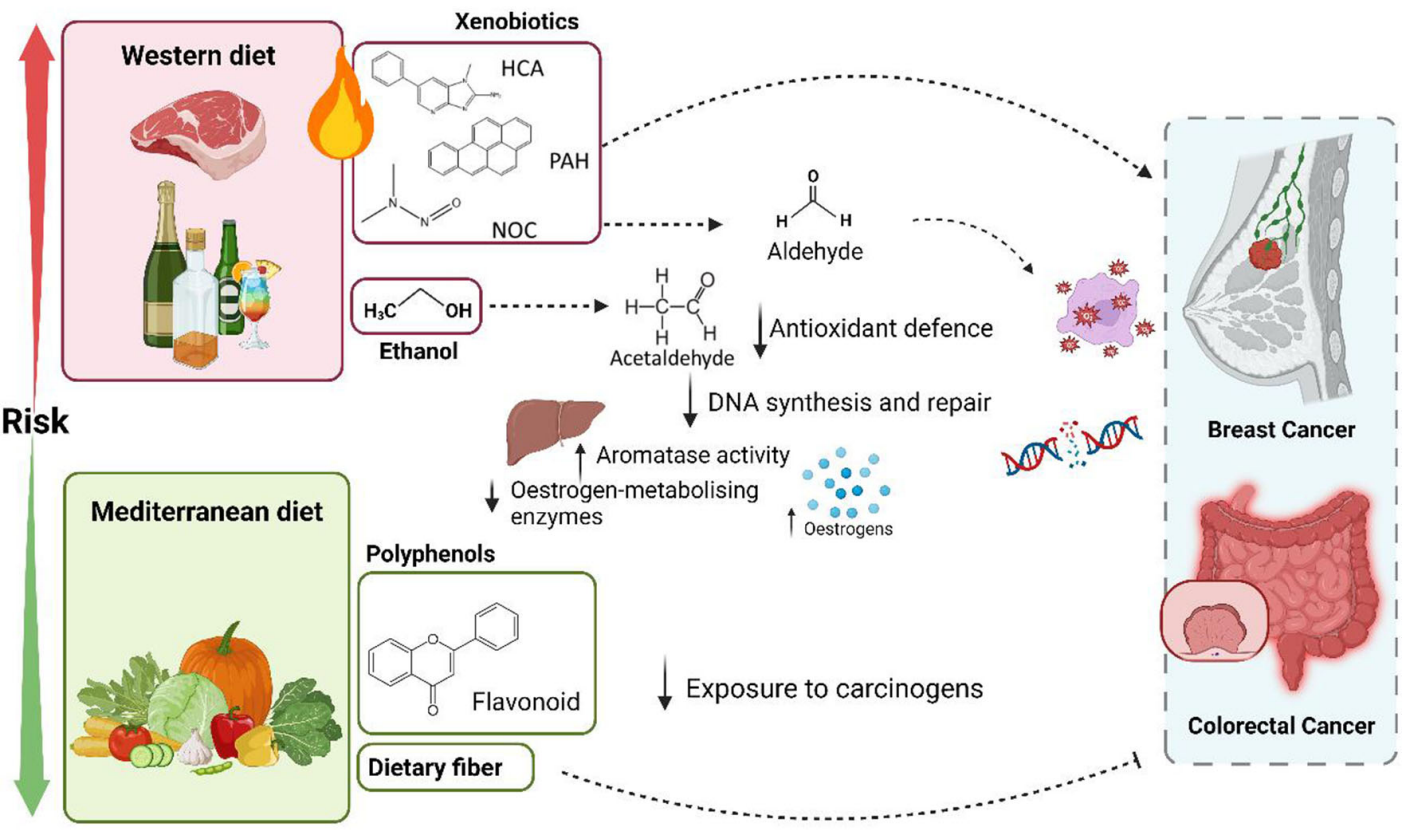
Schematic representation of the main characteristics of the Western diet and Mediterranean patterns that influence the risk of colorectal and breast cancers. Western diets are generally richer in fat, single sugars, processed foods, and red meat and have been associated with higher intakes of ethanol and xenobiotics derived from food processing. Ethanol can be metabolized to acetaldehyde, which impairs antioxidant defence systems and DNA synthesis and repair mechanisms of the host, and interferes with oestrogen-metabolising enzymes and potentiates aromatase activity in the liver, thus increasing circulating free oestrogens. Xenobiotics have mutagenic, genotoxic, and cytotoxic potential. Mediterranean diet is characterized by higher consumption of vegetables, fruits, legumes, extra virgin olive oil, nuts, fish, and whole grains, which lead to a higher intake of polyphenols and fibres that decrease exposure to carcinogens by diverse mechanisms. Figure created with BioRender.com.

Globally, two dietary patterns receive the attention of the scientific community and accumulate the most robust degree of health-related evidence. While the Western pattern is characterized by a high presence of fats, sugars, processed foods, and red meat, the Mediterranean model is represented by a higher consumption of vegetables, fruits, legumes, extra virgin olive oil, nuts, fish, whole grains, and low intake of sugars, red and processed meat and dairy products^[36]^. It is generally recognized that the Westernized pattern is strongly associated with an increased risk of BC and CRC^[37-39]^, while adherence to a Mediterranean diet (MD) has the opposite relationship^[37,38]^ [Figure 1]. MD has been associated with a lower risk of both cancers^[37,40-43]^ and this protective association in BC patients was especially significant in postmenopausal women without red wine consumption^[44]^. Moderate wine intake with meals is a representative element of the MD^[36]^. However, alcohol consumption has been shown to increase the risk of both tumours regardless of the dose^[41,45]^. A consumption of 35-44 grams of ethanol per day is enough to increase the risk of BC by 46% (95% CI = 1*.*33-1.61)^[46-48]^. Even though the promotion of moderate alcohol consumption in the prevention of cancer has been controversial, several hypotheses have been postulated in support of abstaining from alcohol. The specific molecular mechanisms underlying this correlation are still unclear, but it is known that excessive amounts of alcohol can be metabolized to acetaldehyde, interfering in the antioxidant defence system, in DNA synthesis and repair systems^[49]^, as well as in the ability to suppress oestrogen-metabolising enzymes in the liver or by enhancing the aromatase liver activity^[50-53]^. High adherence to a Mediterranean dietary pattern has been associated with decreased CRC risk in several studies^[37]^. The potentially protective effect of MD may be related to lipid metabolism, the protection against oxidative stress, inflammation, and platelet aggregation, or the alteration of hormones and growth factors involved in BC pathogenesis^[54]^, and/or to the potential contribution of other associated healthy behaviours in women who show closer adherence to a MD^[42]^. Furthermore, adherence to healthy dietary and lifestyle patterns may also be associated with improved survival post-diagnosis^[55]^.

Dietary patterns or scores derived from mechanistic approaches have also been studied in association with CRC. For example, the dietary inflammatory index (DII) has been shown to be related to inflammatory markers^[56]^ being consistently associated with an increased CRC risk^[57]^. Conversely, to the promoting role of inflammation, the Non-Enzymatic Antioxidant Capacity (NEAC) index has proven to be a useful tool to estimate the total dietary antioxidant capacity encompassing antioxidants and bioactive compounds present in the diet and their synergistic effects^[58]^.

Concerning individual foods, a lower risk of CRC was noted with a higher consumption of fish and a reduced intake of red and processed meats. This protective effect was limited to fatty fish in BC^[41]^. Red and processed meats have been classified by the International Agency for Research on Cancer (IARC) as “carcinogenic” and “probably carcinogenic” to humans, respectively^[59]^. The exact mechanism by which meat has been related to cancer is unknown. However, the thermal formation of different carcinogens during cooking, such as HCAs and polycyclic aromatic hydrocarbons (PAHs), the endogenous N-nitroso compounds (NOCs) formation from haem iron, the generation of lipid and protein oxidation products, and the addition of NOCs to cured meats may be the underlying causes of this association^[27,60,61]^. In this regard, haem iron found in meat is related to the production of NOCs at the intestinal level and has been associated with the generation of aldehydes with cytotoxic and genotoxic potential^[62]^. PAHs are formed in a large variety of foods, including oils, grains, and vegetables, after applying a heat treatment for cooking (frying, baking, grilling, *etc*.) or processing. Among the different types of PAHs, benzo(a)pyrene (BaP) has been classified as carcinogenic to humans^[63]^. Nevertheless, given the ubiquity of PAHs in food and their presence as environmental contaminants, it is very difficult to assess to what extent the amount ingested from food may contribute to cancer development^[64]^. PAHs can be formed by pyrolysis of organic matter at high temperatures, by direct contact of lipid droplets with a heat source, by the smoke produced during cooking, or by the incomplete combustion of coal or wood in barbecues or grills^[65,66]^. The maximum levels of PAHs have been found in smoked foods and grilled meats^[65]^. HCAs have provided the strongest scientific evidence as cancer risk factors and are the only xenobiotic formed exclusively during the cooking process. HCAs are formed from creatinine, creatine, hexoses, amino acids and some dipeptides, which are present mainly in the muscles of meats and fish^[67,68]^. As the formation of HCAs increases with temperature and with the browning degree of cooked food^[69]^, cooking methods such as frying, grilling, or roasting lead to the formation of higher amounts of HCAs than boiling, stewing, or braising^[70]^. Although most of the existing literature links the intake of these xenobiotics mainly with the risk of CRC, some recent studies have hypothesized that they could also influence BC risk by means of the accumulation of PAHs in breast adipose tissue and their interaction with the cellular oestrogen receptor^[71]^.

Dietary fibre is a dietary component with the potential to decrease the carcinogenic power of ingestion. Consumption of high levels of fibre contributes to reduced exposure of the colon mucosa to carcinogens^[72]^. In addition, fibre acts as an absorbent gel in the faecal mass, making it more difficult for carcinogenic compounds to be incorporated into the organism^[27,73]^.

## GUT MICROBIOTA SIGNATURES IN THE ONSET AND PROGRESSION OF CANCER

### Common features in the alterations of the gut microbiota

The interactions between gut microbiota and cancer onset and progression can be considered at three levels: primary, secondary, and tertiary interactions. Primary interactions refer to the direct association of the microbiota with the tumour microenvironment, by creating dysbiosis that promotes carcinogenesis. The secondary interactions involve the connection between members of the microbial community and changes in the tumour microenvironment in a given tissue/organ. The tertiary interactions are related to the effect of microbial communities on tumours located in different sites in the body^[74]^.

Dysbiosis is a common feature in CRC, and the “driver-passenger” theory has been proposed to explain how gut bacteria named “drivers” induce CRC by progressive damage of the epithelial DNA and then trigger tumorigenesis. In turn, these microorganisms also promote the proliferation of “passenger” bacteria by providing them with a suitable environment for proliferation^[75]^. The substitution of normal microbiota communities by opportunistic pathogen species is a significant and common signature in the gut microbiota of CRC patients. In addition to this, the microbial metabolome and some pro-carcinogenic functions of specific bacteria and fungi also align with the alterations in the microbiota composition. In this respect, alterations in the gut metabolome occur in the early development of CRC^[76]^, and some lipids such as polyunsaturated fatty acids, secondary bile acids, and sphingolipids have been found to be elevated in patients with colorectal adenomas, a previous stage to CRC development^[77]^. It is also interesting to remark that Kim *et al.* found a stronger association between gut microbiota and metabolome in females compared to males^[77]^.

The relationship between gut microbiota and BC is less known. Alterations in gut metagenomes have been reported in postmenopausal BC patients in which the presence of genes encoding for microbial LPS biosynthesis, beta-oxidation, and iron complex transport systems have been detected^[78]^. The pre-existence of gut microbiota disturbances has been correlated with an increased risk of BC metastasis^[79]^. Observational and *in vitro* studies support the relationship between gut microbiota and BC through the travel of metabolites produced by the gut microbiota into the blood to influence BC and immune cells^[80]^. Oestrogens are one of the metabolites that could be influenced by the gut microbiota. The estrobolome is defined as the collection of intestinal bacterial genes that can potentially metabolize oestrogens. The gut microbiota controls levels of oestrogens by producing glucuronidases that deconjugate oestrogens, converting them into active forms. This process could be altered by gut microbiota dysbiosis, thus affecting several conditions such as metabolic syndrome, endometriosis, polycystic ovary, fertility, and cancer, among others^[81]^.

The connection between BC and CRC in women seems to be mediated by elevated levels of sex hormones, although this point is still controversial. Some researchers have identified an increased risk of CRC or the occurrence of adenomatous polyps in patients with a history of BC^[26,82]^.

### Colorectal cancer

**Figure 2:**
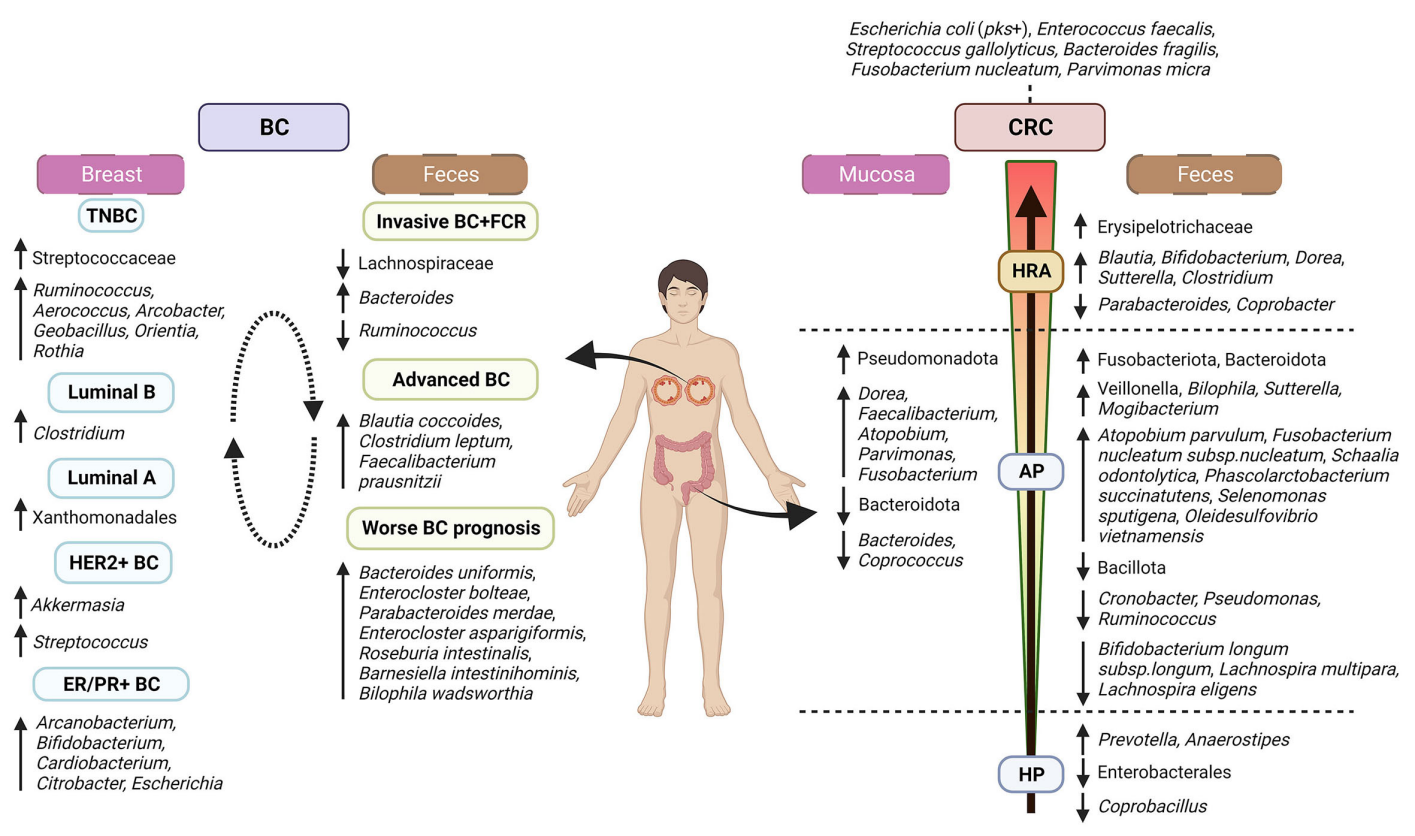
Schematic representation of the microbial group associations with CRC and BC. Differences in microbial composition and abundance are indicated according to the type of sample (stool, breast, or intestinal biopsies), the stage of cancer development, the molecular characteristics of the disease, and the approach/design of the study. The left side of the image shows breast microbiota profiles in different BC subtypes by 16S rRNA gene sequencing and pan-pathogen array (Pathochip array) and gut microbiota patterns associated with BC stage and prognosis by 16S rRNA gene and shotgun metagenomics. The right side shows differences in the intestinal mucosa microbiota detected by 16S rRNA gene sequencing, terminal restriction fragment length polymorphism, clone sequencing, and fluorescent in-situ hybridization analysis of the 16S rRNA genes for patients diagnosed with intestinal adenomatous polyps. Faecal samples were studied using 16S rRNA gene sequencing for patients diagnosed with HP and shotgun metagenomics and 16S rRNA for patients showing AP and high-risk adenomas (HRA: presence of high-degree dysplasia, tumours ≥ 10 mm, and/or presence of three or more adenomas of any size). Up or down arrows indicate higher or lower presence, respectively, of the microbial groups. The information represented has been obtained from studies showing significant changes in CRC^[76,85,86,95-103]^ and BC processes^[115-117,123,124]^. AP: Adenomatous polyps; BC: breast cancer; CRC: colorectal cancer; ER: oestrogen receptor; FCR: fear of cancer recurrence; HER2: human epidermal growth factor receptor 2; HP: hyperplastic polyps; HRA: high-risk adenomas; PR: progesterone receptor; TNBC: triple-negative breast cancer. Figure created with BioRender.com.

The identification of gut microbiota signatures through CRC development is a challenging task that would need systems biology approaches for its development; this could be of interest for its application to the prevention of disease progression and for the potential discovery of new biomarkers. Despite the variable genetic basis of the disease, late-onset CRC, which is the most commonly detected, is highly influenced by environmental factors, such as lifestyle and diet^[83,84]^. Microbiological key signatures may vary according to the different stages of the disease, thus influencing its progression. In terms of microbial diversity, values of this parameter decrease when healthy patients are compared with CRC cases^[85-88]^, and also shift when late CRC samples (Stages III and IV) are compared with early CRC samples (Stages I and II)^[89]^. CRC is accompanied by dysbiosis, and the presence of certain bacteria has been strongly associated with CRC development at any point of its progression. Among them, *Escherichia coli* (*pks*+), *Enterococcus faecalis*, *Streptococcus gallolyticus* (previously known as *Streptococcus bovis*), *Bacteroides fragilis*, *Fusobacterium nucleatum* or *Parvimonas micra* are frequently found increased in faecal and tumour CRC samples^[85,86]^ [Figure 2]. Differences in the microbiota of patients according to the type of sample analysed should also be considered, i.e., biopsies or faecal samples. In this regard, paired samples from mucosal tumour tissue and surrounding non-tumour mucosa were similar in terms of microbial taxa and overall composition, according to various multiple cohort studies^[89-92]^. Moreover, a study by Flemer *et al.* revealed that the signatures detected in mucosa and tumour samples of CRC patients compared to samples from healthy volunteers were still evident in faecal samples^[93]^. In spite of these findings, it is difficult to ascribe the cause of dysbiosis to one or more microorganisms. As right and left colon cancer present distinct molecular signatures, the location of the tumour can contribute to variations found in the gut microbiota among individuals. In this way, a differential higher presence of genera *Fusobacterium*, *Escherichia*, and *Leptotrichia* has been reported in the mucosa-associated microbiota of the left colon, whereas *Prevotella*, *Selenomonas*, and *Peptostreptococcus* were more abundant in samples from right colon^[94]^. Nevertheless, many studies lack information about the anatomical location of tumours, which increases the difficulty of comparing them and reaching sound conclusions.

The earliest stages of mucosa alterations, before reaching the status of CRC, are characterized by the presence of a certain grade of gut mucosa damage and/or alterations, such as the presence of polyps. Some of the alterations showing lower malignant potential are classified as hyperplastic polyps (HP), which belong to the serrated neoplasia pathway. When the faecal microbiota of healthy individuals was compared with that of subjects showing HP, an increased abundance of genera *Prevotella* and *Anaerostipes* and decreased abundance of the order Enterobacterales and the genus *Coprobacillus* was found in the HP group with respect to control samples^[95,96]^.

The presence of adenomatous alterations, developed through the adenomatous pathway, represents a medium risk for the development of CRC, and therefore, the surgical removal of adenomas after their diagnosis is a desirable prevention procedure. In patients showing adenomatous polyps (AP), a decreased and increased relative abundance of faecal levels of phyla Bacillota (previously known as Firmicutes) and Fusobacteriota, respectively, was detected compared with faecal samples from healthy controls^[97]^. This was consistent with the observations by Hale *et al.*, who reported a differential increased abundance of Bacillota and the *Veillonella* genus in faeces from the control group. Moreover, the adenoma group of this study showed a differentially increased abundance of the phylum Bacteroidota and *Bilophila*, *Sutterella* and *Mogibacterium* genera^[98]^. In contrast, the study carried out by Shen *et al*. in mucosa samples reported a lower abundance of Bacteroidota and a higher abundance of Pseudomonadota (former Proteobacteria) in adenoma cases as compared to the control group. At the genus level, case subjects showed an increased abundance of *Dorea* sp. and *Faecalibacterium* sp., and lower proportions of *Bacteroides* sp. and *Coprococcus* sp. than controls^[99]^. Several members from the class Clostridia and some genera such as *Cronobacter*, *Pseudomonas*, and *Ruminococcus* were found to be reduced in the stools of individuals with conventional adenomas compared to faeces from controls^[96,100,101]^. Furthermore, the study by Yachida *et al*. revealed nine species shifted in faecal samples of subjects with multiple AP. These include enriched levels of *Atopobium parvulum*, *Fusobacterium nucleatum* subsp. *nucleatum*, *Schaalia odontolytica*, *Phascolarctobacterium succinatutens*, *Selenomonas sputigena*, and *Oleidesulfovibrio vietnamensis*, and a decrease in *Bifidobacterium longum* subsp. *Bifidobacterium longum* subsp. *longum*, *Lachnospira multipara*, and *Lachnospira eligens* as compared to samples from healthy individuals^[76]^. Interestingly, similar shifts were found by Saito *et al.*, who found a significant increase in *Atopobium*, *Parvimonas*, and *Fusobacterium* in mucosal colorectal adenoma samples as compared to healthy mucosa samples^[102]^.

As malignant transformations occur, AP may turn into high-risk adenomas (HRA). Faecal samples of individuals presenting HRA displayed an increased relative abundance of Erysipelotrichaceae and the genus *Blautia*^[95]^. Indeed, this genus was also found enriched in advanced adenoma faecal samples in another study conducted by Xu *et al.*, along with increased abundances of *Bifidobacterium*, *Dorea*, *Sutterella*, and *Clostridium* whereas *Parabacteroides* and *Coprobacter* were enriched in controls^[103]^.

An increased risk of CRC is also associated with the development of less frequent sessile serrated adenomas (SSA) from the serrated pathway. Stools from subjects with SSA showed a decreased abundance of class Erysipelotrichia compared to samples from control subjects^[96]^.

On the other hand, the presence of aberrant crypt foci (ACF) is the first histological alteration of the intestinal mucosa. Although these elements are not currently considered in clinical diagnosis, their evolution is being followed in CRC intervention studies with experimental animals^[104]^. Some of these studies show that the oral administration of probiotic strains such as *Lacticaseibacillus rhamnosus* or *Butyrivibrio fibrisolvens* attenuates the formation of ACF in experimental animals^[105,106]^. In a similar way, possible early microbiota alterations associated with ACF in humans could constitute an interesting field of research in the area of CRC.

Shifts in the microbiota abundance at the early stages of development of intestinal mucosal damage may be the cause of a plethora of changes in human metabolism, thus influencing health. For example, a disturbance in the gut microbiota composition could be associated with lower faecal SCFA concentrations, finally decreasing the production of IgA^[88]^. Additionally, the activity of gut microbiota is involved in the production of some bile acids such as deoxycholic, glycocholic, and taurocholic acids, which have been found to increase in CRC-related conditions^[76,107,108]^. Moreover, in CRC-diagnosed patients, higher faecal concentrations of aromatic and branched-chain amino acids or polyamines such as putrescine and cadaverine are usually associated with some microbial groups^[76,107-109]^. Furthermore, certain bacteria are producers of toxic metabolites or genotoxins^[110]^. Various studies have found differential associations between microbes and metabolites in CRC for members of the Bacteroidota, Bacillota, and Actinomycetota phyla or even families such as Enterobacteriaceae^[77,111]^. In this regard, studies on how faecal microbiota and metabolites are intertwined at the early stages of intestinal mucosa damage are still scarce.

### Breast cancer

In the last years, the microbiota has gained prominence as an important regulator of tumour incidence and progression as well as tumour microenvironment in BC, but it is currently unknown whether cancer pathology leads to alteration in the gut microbiota or whether dysbiosis in itself is the carcinogenic factor. The study of the interaction between the metagenome and the host genome has been the subject of investigation and could be instrumental in greatly improving the mechanistic understanding of variable outcomes among different patients. Unfortunately, the role of microbiota in BC is only starting to be revealed and current data is controversial because the different clinical studies have been performed with a small sample size and, in most of the cases, are cross-sectional. In addition, clinical studies present patients with different ages and tumour stages and sometimes there is not a control group, which frequently makes the results not comparable.

Regarding the differences in gut microbiota between women with and without BC, the current data is not clear and the availability of clinical studies is limited. Goedert *et al*. reported that postmenopausal women with BC have altered faecal microbiota composition and oestrogen-independent lower alpha diversity^[112]^. The opposite results were indicated by Zhu *et al*., who observed that BC patients displayed higher microbiota richness and diversity; however, it is important to note that the two above clinical studies have not considered the different types of cancer^[78]^. A recent observational study has not found gut microbiota differences in women with and without BC from Ghana^[113]^. Likewise, it has also examined the gut microbiota from postmenopausal women with ER+/HER2- BC and postmenopausal controls, and no differences were observed in intestinal microbiota richness, diversity, and composition^[114]^. The microbiota-gut-brain axis in BC patients has also been recently studied. It has been observed that women diagnosed with invasive BC and associated with fear of cancer recurrence have a different microbial profile characterized by lower microbial diversity, higher relative abundance of *Bacteroides*, and lower relative abundance of Lachnospiraceae and *Ruminococcus*^[115]^ [Figure 2].

It seems that the intestinal microbiota is altered in advanced BC and that alterations in the microbiota may affect BC progression. Luu *et al.* concluded that gut microbiota differs according to the type of tumour and BMI. They indicated that patients with advanced stage of BC had increased numbers measured by qPCR of *Blautia coccoides* and *Clostridium leptum* clusters, as well as *Faecalibacterium prausnitzii*, compared with patients with low-grade cancer^[116]^. It has been indicated by shotgun metagenomics that healthy volunteers and pre and post-treatment samples of patients with low risk or early BC (mostly RH+ patients) were enriched in *Butyrivibrio crossotus* and *Collinsella aerofaciens* but on the contrary *Bacteroides uniformis*, *Enterocloster bolteae*, *Parabacteroides merdae*, *Enterocloster asparagiformis*, *Roseburia intestinalis*, *Barnesiella intestinihominis* and *Bilophila wadsworthia* were associated with worse BC prognosis^[117]^.

When considering microbiota dysbiosis and the association of the microbiota with BC development, the breast’s microbiome also has to be taken into account. Evaluating the gut and the breast microbiota in the same patient is very useful and can provide associations between the microbiota and BC systematically. The human breast is not sterile and the microorganisms could come from the gut, the skin, via the nipple-areolar orifices, nipple-oral contact via lactation, and/or sexual contact^[118,119]^. The comparison of breast tissue from invasive and benign BC and healthy volunteers also revealed differences in their microbiota^[120-122]^. It is quite consistent between different studies that the dominant bacterial phyla in breast tissues are Pseudomonadota, followed by Bacillota and Actinomycetota, but as it occurs with the gut microbiota, opposite bacterial profiles have been reported between patients with and without BC in different studies. Two recent works have explored the differences in local microbiota between different molecular types of BC (luminal A, B, HER2+, and TNBC) and identified different bacterial profiles associated with each type of BC^[123,124]^. TNBC was, for example, more abundant than the other types of BC in phyla Euryarchaeota, Cyanobacteria, and Bacillota^[124]^. Cross-sectional studies cannot determine causality, and in addition, the comparison of breast microbiome among clinical studies is difficult because of differences in tissue source, the experimental protocols used for DNA extraction, and microbiota analysis. However, standardizing this type of study could help to improve our knowledge of the relationship between microbiota and BC and open the possibility of assessing prognoses and identifying bacteria associated with procarcinogenic stages that could allow suggesting breast microbial interventions (diet, antibiotics, selective antibiotics...) as an adjuvant to the standard therapies.

There is emerging evidence suggesting that the gut microbiota may also impact BC and its medical treatment by mediating drug efficacy and toxicity. The estrobolome, as indicated before in this review, may contribute to explaining the relationship between gut microbiota and hormone-dependent BC. Oestrogens can influence the gut microbiome, and in its turn, the microbiota can significantly affect oestrogen levels through microbial β-glucuronidase and β-glucosidase enzymes able to deconjugate oestrogens in the intestine, enabling these hormones to bind to receptors of eukaryotic cells and leading to the subsequent physiological downstream effects^[125]^. Gut microbiota genera that can express β-glucuronidases, include *Alistipes*, *Bacteroides*, *Bifidobacterium*, *Citrobacter*, *Clostridium*, *Collinsella*, *Dermabacter*, *Edwardsiella*, *Escherichia*, *Faecalibacterium*, *Lactobacillus*, *Marvinbryantia*, *Propionibacterium*, *Roseburia*, and *Tannerella*. Additionally, it has been reported that a decrease in the gut bacterial diversity could lead to oestrogen release and finally an increase in the BC risk^[126]^. Microbial β-glucuronidase could also deconjugate xenobiotics and xenoestrogens, increasing the time they remain in the organism^[127]^. The influence of all these known factors, including the complexity of the microbiota, supports the suggestion that these types of cancers are initiated and developed by multiple pathophysiological factors. Radiotherapy, chemotherapy, and immunotherapy can also modify the microbiota, and at the same time, the gut microbiota can metabolize medical drugs in systemic treatments of cancer, modulating the immune response to treatment and influencing the development of side effects of therapies. However, investigation in this way is still scarce^[115,128]^.

## MICROBIOTA AND DIET INTERACTIONS IN THE INITIATION AND PROGRESSION OF CANCER

Recent studies have evidenced a high correlation among habitual diets, microbiome composition and its associated metabolome, health and disease markers, and host metabolism^[129-131]^. Generally, diets that show higher diversity and higher scores in health parameters, such as the MD, display modified ratios of specific bacteria, with elevated levels of microorganisms producing SCFAs and lower cancer risk. In contrast, less healthy diets, such as Western patterns, promote specific bacteria that may affect carcinogenic pathways, reduce intestinal epithelial immune defences, and alter intestinal permeability^[132]^. Some microbial groups have been positively associated with healthy plant-based diets and negatively with metabolic-risk markers, which mostly include butyrate producers such as *F. prausnitzii*, *Prevotella*, *Roseburia hominis, Agathobaculum butyriciproducens,* and *Anaerostipes hadrus*, as well as *Akkermansia muciniphila,* among others^[129,131]^. Notably, De Filippis *et al.* reported that microbes poorly characterized until now drive the strongest associations between microbiome and habitual diet, which can be mostly due to the difficulty in cultivating these microorganisms^[129]^. This emphasizes the need for culturomics and metabolomics approaches to improve our knowledge of these potentially beneficial uncultivable microorganisms. In contrast, some microbial species were positively associated with less-healthy plant-based and animal-based diets and include, among others, several *Clostridium* species, *Mediterraneibacter gnavus* and *Flavonifractor plautii,* with the latter two also associated with CRC^[133,134]^.

The gut microbiome plays a key role in the interplay between diet and host health. The gut microbiome and the human intestinal immune system are interconnected. In a healthy status, pathogens are suppressed or remain compartmentalized in the gut, and the intestinal barrier efficiently prevents the passage of toxic components from the intestine to the general circulation. On the other hand, a low inflammatory and hyporesponsive environment is generated, and there is a downregulation of reactive immune T-cells^[135-137]^.

A role as preventive agents against cancer has been attributed to some dietary compounds, among which are fibres and phytochemicals such as phenolic compounds/polyphenols. Fibres are indigestible complex carbohydrates that represent a major component of plant-based diets. When these compounds reach the colon, some microorganisms of the gut microbiota are able to use them as fermentable substrates, producing SCFAs. Acetate, propionate, and butyrate interact with intestinal epithelial cells, can cross the epithelial layer and interact with immune cells of the lamina propria, and can enter into systemic circulation to reach other tissues. SCFAs can serve as cell energy sources and display anti-inflammatory properties through G protein-coupled receptors (GPCRs) and through the increase of histone hyperacetylation, which promotes the immune-mediated apoptosis of cancer cells. In the intestine, SCFAs participate in microbial cross-feeding interactions, promoting beneficial modifications of the intestinal microbiota. Moreover, dietary fibres contribute to shortening the intestinal transit time and sequestrating dietary toxic molecules^[138,139]^, which helps to decrease intestinal toxicity. Several meta-analyses suggest that individuals consuming higher amounts of dietary fibre may benefit from a reduction in the incidence of CRC and a small reduction in the incidence of BC^[140]^.

Most dietary phytochemicals are present in nature as glycoconjugates and can be transformed into aglycones through deglycosylating enzymes produced by the intestinal microbiota, then increasing their biological activity and bioavailability. Deconjugated compounds can be absorbed or be further metabolized by the gut microbiota into other products. This biotransformation of phenolic compounds is subjected to a high interindividual variability due to differences in the intestinal microbiota composition, which then influences the final effect of phenolic compounds on the host^[139]^. Soya isoflavonoids are transformed into aglycones and further metabolized to products such as equol, a compound derived from daidzein^[141]^ that presents the greatest antioxidant activity among isoflavones. The best-characterized microorganism to date involved in the production of equol is *Adlercreutzia equolifaciens* (family Eggerthellaceae, class Coriobacteriia, phylum Actinomycetota)^[142]^. Ellagitannins can be transformed into urolithins, compounds that display enhanced biological activity with respect to the untransformed ones^[143]^. Some species of the genus *Gordonibacter* (family Eggherthellaceae) have been found to be related to the production of urolithins from ellagic acid^[144]^. Plant lignans are mostly converted into mammalian enterolignans enterodiol and enterolactone^[145]^. These derived phenolic compounds have a variable capacity to inhibit angiogenesis and inflammatory factors, which is related to their anticancer properties^[139]^. The conversion of lignans occurs in several steps in which diverse species from *Bacteroides, Clostridium, Butyribacterium, Eubacterium, Peptostreptococcus*, *and Blautia* are involved. Members of *Eggerthella lenta* and *Eggerthella* sp., *Clostridium scindens*, and *Ruminococcus* sp. carry out the final steps in the formation of enterodiol and enterolactone^[146]^. Lignans and soya isoflavonoids belong to the phytoestrogens group of compounds and can exhibit estrogenic and antiestrogenic activities in humans depending on the levels of the human hormone estradiol^[147]^. High intake of lignans and isoflavones have been associated with a reduced risk of BC, although in the case of soy, evidence suggests this consumption must occur preferentially early in life, during childhood and/or adolescence^[148]^. Nevertheless, some *in vitro* studies have reported an inhibitory effect of the isoflavone genistein on the aromatase inhibitor Fadrozole, a pharmacological agent used for the treatment of oestrogen-responsive BC^[149]^. In this regard, the report of some controversial results relating food or soy supplement consumption and markers of BC^[150,151]^ prompted more observational studies supporting the positive effect of soy isoflavone intake for BC post-diagnosis^[148,152]^. The study of the role played by diet in BC has an added difficulty, because the mass effect of the two microbiotas, from gut and breast, should be considered. In the meantime, more studies are needed to disentangle the microbiota-mediated association between BC and CRC in women.

NOCs, HCAs, PAHs, and acrylamide that could be present in diet after cooking and processing foods^[27]^ can promote modifications in the intestinal microbiota profiles. The intestinal microbiota can metabolize and transform them, increasing or decreasing their toxicity to a variable extent depending on the microbiota composition, the amount and type of compound, other components of diet interacting with microbiota and xenobiotic, and the detoxification mechanisms of the host (cytochrome P450 enzymes superfamily)^[27]^. Moreover, although dietary ethanol does not enter direct contact with the microbiota, it can unfavourably modify it through the activation of gut microbial acetate dissimilation pathways^[153]^. Dietary xenobiotics can alter the microbiota composition and its metabolic activity as well. For a more comprehensive review of this subject, we have recently outlined the interactions of xenobiotics derived from food processing with the intestinal microbiota in the context of CRC^[27]^.

In spite of recent advances in this matter, most microorganisms and microbial biochemical reactions mediating the transformation of dietary bioactive compounds and xenobiotics by the gut microbiota remain unknown, which represents a challenging field of research connecting the human microbiota, diet, and prevention and treatment of BC and CRC. The role played by micronutrients as phenolic compounds and phytochemicals and dietary carcinogens has proven difficult to study due to the small quantities of these agents in human diets. In this regard, integrated research including experimental models and germ-free mice could be useful in the study of the role that diet, especially in the case of micronutrients and carcinogens, plays in the alteration of the microbiota and its connection with CRC and BC.

### Translational aspects

Diet is one of the main modulators of gut microbiota. SCFA and other microbial metabolites are synthesized by members of the gut microbiota from different dietary sources, which determine the differential growth of certain microbial populations. Dietary patterns and specific food and nutrients exert pleiotropic systemic effects in the body, also influencing the physiology and immune system of the host. Therefore, dietary interventions focused on modulating the gut microbiota could contribute to preventing the development of cancer, slowing down its progression, and/or enhancing the efficacy of anticancer therapies. Possible translational approaches in this way can be directed to interventions with diets with low DII, and high NEAC index. Specifically, more translational research is needed to know the short-term, medium-term, and lasting effects of the reduced intake of dietary xenobiotics and the increased consumption of fibres and phytochemicals in CRC and BC. The use of probiotics, prebiotics, and postbiotics may have a protective role in cancer prevention^[21]^. Moreover, addressing dysregulated microbiota-gut-brain axis communication is an area of expansion for research and personalised cancer care, including personalised or adapted diets and the potential use of psycobiotics^[34,154]^.

## CONCLUSION

The scientific evidence underlined in this review on the interconnection between diet and the intestinal microbiota in the evolution of precancerous lesions towards BC and CRC points to the need for deeper studies to design dietary strategies for cancer prevention. More studies, including systems biology and the integration of experimental models and germ-free mice, are required for the study of the role of the less abundant dietary components as carcinogens and micronutrients in the development of BC and CRC. It is necessary to decipher the microbiota-mediated association existing between BC and CRC in women, considering the two interacting microbiotas from gut and breast. Most microorganisms and biochemical pathways that mediate the transformation of dietary bioactive and harmful compounds by the gut microbiota remain unknown. In spite of variations found in some microbial populations during the progression of mucosal lesions to cancer, its true role in the development of the disease is not clear. It seems then reasonable to conclude that neoplasia may be triggered by the net influence of a wide variety of microbes that provide a microclimate suitable for the malignant change when operating in genetically susceptible and environmentally inducing conditions. Therefore, there is an urgent need to improve our knowledge in this field in order to design strategies for the treatment and prevention of BC and CRC through the binomial diet-microbiota. Current advances in omics techniques (Next Generation Sequencing, culturomics, and metabolomics) and bioinformatics are available to address this complex task.

## DECLARATIONS

### Copyright

© The Author(s) 2023.
